# Improving Treatment Outcome in Children With Obesity by an Online Self-Control Training: A Randomized Controlled Trial

**DOI:** 10.3389/fped.2021.794256

**Published:** 2021-12-23

**Authors:** Eline Vermeiren, Tiffany Naets, Annelies Van Eyck, Leentje Vervoort, Marijke Ysebaert, Nele Baeck, Ann De Guchtenaere, Maria Van Helvoirt, Ann Tanghe, Luc Bruyndonckx, Benedicte Y. De Winter, Stijn L. Verhulst, Kim Van Hoorenbeeck, Caroline Braet

**Affiliations:** ^1^Laboratory of Experimental Medicine and Pediatrics, University of Antwerp, Antwerp, Belgium; ^2^Department of Developmental, Personality and Social Psychology, Ghent University, Ghent, Belgium; ^3^Department of Pediatrics, Antwerp University Hospital, Edegem, Belgium; ^4^Department of Developmental Psychology, Radboud University, Nijmegen, Netherlands; ^5^Department of Pediatrics, Jan Palfijn Gent Hospital, Gent, Belgium; ^6^Zeepreventorium VZW, De Haan, Belgium; ^7^Department of Pediatric Cardiology, Amsterdam University Medical Centers, Amsterdam, Netherlands

**Keywords:** childhood obesity, treatment outcome, BMI reduction, self-control, attention, inhibition, executive functions, randomized controlled (clinical) trial

## Abstract

**Background:** Currently available treatment programs for children with obesity only have modest long-term results, which is (at least partially) due to the poorer self-control observed within this population. The present trial aimed to determine whether an online self-control training, training inhibition, and redirecting attentional bias, can improve the short- and long-term treatment outcome of (in- or outpatient) child obesity treatment programs.

**Methods:** In this double-blind multi-center randomized controlled trial (RCT), participants aged 8–18 years with obesity were allocated in a 1:1 ratio to receive an online self-control or sham training added to their in- or outpatient multidisciplinary obesity treatment (MOT) program. The primary endpoint was BMI SDS. Data were analyzed by linear mixed models and the main interactions of interest were randomization by time and randomization by number of sessions, as the latter was cumulatively expressed and therefore represents the effect of increasing dose over time.

**Results:** One hundred forty-four inpatient (mean age 14.3 ± 2.2 years, BMI 2.7 ± 0.4 SDS, 42% male) and 115 outpatient children (mean age 11.9 ± 2.1 years, BMI 2.4 ± 0.4 SDS, 45% male) were included. Children's BMI lowered significantly during treatment in both the in- and outpatient treatment centers, *p* < 0.001. In a mixed model with BMI as dependent variable, randomization by time was non-significant, but the number of self-control trainings (randomization ^*^ number of sessions) interacted significantly with setting and with age (*p* = 0.002 and *p* = 0.047), indicating a potential effect in younger inpatient residents. Indeed, a subgroup analysis on 22 inpatient children of 8–12 years found a benefit of the number of self-control trainings on BMI (*p* = 0.026).

**Conclusions:** The present trial found no benefit of the self-control training in the entire study population, however a subgroup of young, inpatient participants potentially benefited.

## Introduction

Childhood obesity forms one of the largest public health challenges of the twenty-first century and currently a lifestyle intervention to decrease dietary intake and increase physical activity remains the cornerstone of treatment (1). Both, in- and out-patient programs, have proven effective in reducing BMI (1, 2), although weight regain frequently occurs (3, 4).

One contributor to these modest long-term outcomes is the poor self-control in children with obesity, leading to decreased behavioral control (5). As stated in the Dual Pathway model, self-control results from bottom-up reactivity regulated by top-down executive functioning (6). Bottom-up reactivity comprises the automatic, habit-driven responses toward stimuli in the environment (7). One bottom-up process is attention, and “attention bias” refers to how salient stimuli with high motivational or affective value quickly grasp attention (8). Top-down executive functions are neuropsychological control processes initiating goal-directed behavior and overcoming automatic reactions to external environmental stimuli (9). Executive functioning encompasses different cognitive control tasks, such as inhibition, cognitive flexibility, and working memory, which are all involved in successful self-control (9, 10).

Generally, in subjects with obesity an imbalance exists between an increased bottom-up reactivity, reflected by an attentional bias toward (unhealthy) foods (11), and lowered top-down inhibitory control to counteract automatic reactions on environmental stimuli, reflected by being unable to resist palatable food even in the absence of hunger (12). Previous research in children with obesity has associated poor self-control with less weight loss during treatment (13) and more difficulties maintaining weight loss thereafter (14). Therefore, strengthening self-control of children engaging in obesity treatment seems highly indicated to improve the short- and long-term treatment outcome.

Fortunately, experimental lab studies in adults with obesity show the potential of self-control training by tempering attention bias (8) or increasing inhibition (15) as do some pediatric studies on obesity (16–18). This rationale led to the development of the “WELCOME” trial, which stands for “improving WEight controL and CO-Morbidities in children with obesity via Executive function training.” The present trial explored the effect of a self-control vs. a sham training added to a multidisciplinary obesity treatment (MOT) program. This is the first randomized controlled trial (RCT) assessing the “real-world” effectivity (as opposed to the “in-lab evaluation”) of a combined self-control training (training both inhibition and attention) across different treatment settings (e.g., in- and out-patient) in a large sample of treatment-seeking children with obesity distributed over a broad age range (8–18 years). Furthermore, the present trial evaluated both short- and long-term BMI outcome and self-control.

We hypothesized that

1) The group with the self-control training would have a better BMI outcome compared to the group with the sham training. Here, effects of interest are:
  – the interaction of randomization (self-control vs. sham training) *by* time
  – the interaction of randomization (self-control vs. sham training) *by* number of sessions, as the latter incorporates the impact of the dose–response relation over time as “number of sessions” is cumulatively expressed.

The decision to incorporate the number of sessions was based on a recent meta-analysis indicating that the number of sessions modulated the training effect (19). Furthermore, age, gender, and treatment setting could likely influence the training effects and are therefore incorporated and controlled for in the analyses.

2) The training, when effective on BMI reduction, would result in training-related improvements in self-control. We specifically look at training-related improvements in self-control, as MOT itself improves behavior control (5).

## Methods

### Study Design

**Figure 1:**
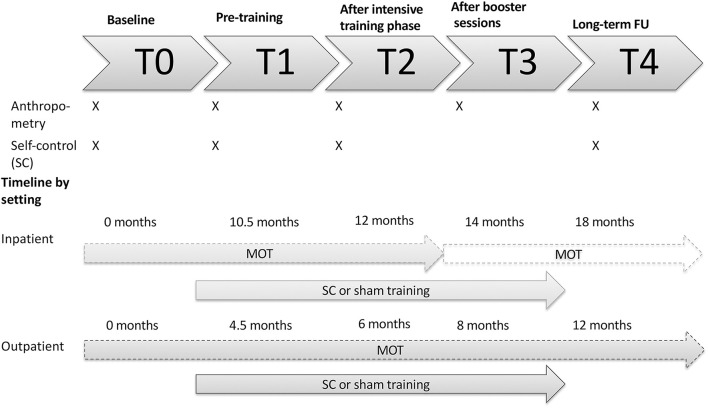
Visual representation of the study design by setting. An overview of which assessments are performed at each timepoint is provided. T, timepoint; MOT, multidisciplinary obesity treatment; FU, follow-up; SC, self-control.

A double-blind multi-centered RCT was conducted to objectify the added value of a bottom-up and top-down self-control training on top of the currently existing MOTs. Randomization was performed by an online program [QMinim (20)]. Patients were randomized by a 1:1 allocation rate based on pretreatment age, gender and BMI SDS to receive either the self-control or the sham training. The training was added after the second study visit (T1) as depicted in Figure 1.

The study was approved by the Ethics Committees of the University Hospital of Antwerp and Ghent (EC n°B670201731779) and was performed according to the principles of the declaration of Helsinki. The trial was registered at the ISRCTN register (n°ISRCTN14722584). Written informed assent/consent was obtained from the patient and their caregiver before the start of the study.

### Participants

Children aged 8–18 years old were recruited between July 2017 and January 2020 upon admission to an in- or outpatient childhood MOT (T0), if they were diagnosed with obesity based on the criteria of the International Obesity Task Force based on the Flemish growth charts (21, 22). Patients were excluded in case of a genetic or endogenous origin of the obesity or in case of simultaneous participation in another interventional trial.

### Multidisciplinary Obesity Treatment

All participants engaged in a standard MOT aimed at reducing BMI by altering dietary patterns and increasing physical activity, combined with (cognitive) behavioral change techniques and parental involvement.

In the inpatient setting, participants with severe obesity and comorbidities entered a 12-month MOT at a pediatric rehabilitation center “Het Zeepreventorium” (ZPM) (De Haan, Belgium). Briefly summarized, the multidisciplinary program is targeted at reducing BMI by increasing physical activity up to a minimum 10 h a week and decreasing caloric intake by implementing a healthy diet according to sex and age in a highly structured environment. Additionally, psychological and contextual support is offered individually and in group. The caregivers are invited for sessions where parenting styles are discussed and education on a healthy lifestyle is offered simultaneously. This program has been elaborately discussed elsewhere (23).

In the two outpatient settings, the Antwerp University Hospital (UZA) and the Ghent Jan Palfijn Hospital (JPG), participants with mild to moderate obesity were included upon entrance of an MOT, where (similar to inpatient treatment) a dietician and pediatrician (and if required a psychologist) are involved. The dietician works on a step-by-step approach to establish a sustainable healthy diet, whereas the pediatrician monitors the evolution of the obesity severity and initiates treatment for related co-morbidities if present. Psychological support can be provided on request. Throughout the sessions, physical activity with a minimum of 1 h/day is highly encouraged. Patients are followed clinically as long as required based on their BMI and obesity-related comorbidities.

### Self-Control Training

**Figure 2:**
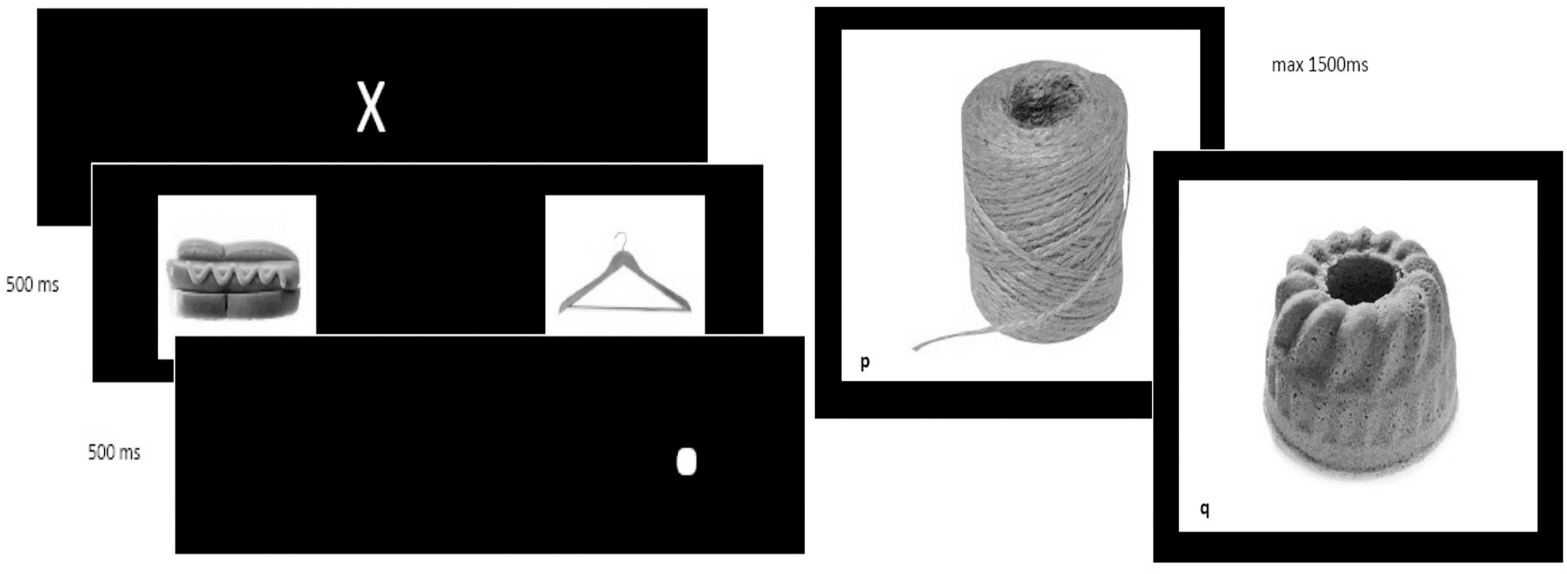
Example of the dot-probe and Go/No-Go task. (Left) Example of the dot-probe task, used to redirect bottom-up attentional bias and (Right) go and no-go trial in the Go/No-Go task used to train top-down inhibitory control.

The self-control training consisted of a computer training containing a bottom-up attention training and a top-down inhibition training, an example of both is depicted in Figure 2.

For the attention training, the Dot Probe task was used (24). First, a white cross was shown. Subsequently, two stimuli (food or neutral objects) were shown and followed by the appearance of a white dot at the location of the stimulus when these pictures were removed. Patients were asked to locate the dot and press “e” or “i,” when the white dot was presented on the left (“e”) or right (“I”) side of the screen. In congruent trials, the dot was located at the same side of the salient (food) stimulus. In incongruent trials, the dot was located at the opposite side (at the location of the neutral stimulus). In the self-control training, 90% were incongruent trials where the dot appeared at the opposite side of the food stimulus, aiming to reduce this selective attentional bias by presenting the dot on the opposite side of the screen as where the “attention drawing” cue is presented (24). This ratio was 50/50 in the sham training.

The inhibition training relied on a Go/No-Go (GNG) task (25), where a picture of a neutral or unhealthy food stimulus was accompanied by a “go” or “no-go” cue (in this case a letter, p or q). Rapid responses were evoked in “go trials,” in which patients needed to respond by pressing the spacebar as fast as possible when the “go”-stimulus was presented. In “no-go trials,” patients were required not to react. In the experimental training, 90% of the “no-go” trials were accompanied by a picture of unhealthy food, aiming to restrain impulsive responses toward unhealthy food stimuli (25). In the sham training, only 50% of the no-go trials were accompanied by an unhealthy food picture (and 50% with a neutral stimulus).

The online self-control training was provided in two phases: the intensive training phase and the booster phase (see Figure 1). The intensive phase was introduced after T1. Here, patients were required to complete 12 sessions, two sessions a week for 6 weeks until T2. After T2, booster sessions were started at a frequency of one session a week for 8 weeks, till T3. Every training session had a duration of 30 min. In a final follow-up (T4) weight and length were collected.

The training software was developed by ImplicitMeasures.com (Ghent, Belgium).

### Outcome Measures

The primary endpoint was BMI SDS. To rule out a possible underestimation of changes in BMI with BMI SDS in children with severe obesity (26), we have repeated the main analysis with BMI expressed as a percentage of the 95^th^ percentile as recommended by Freedman et al. (27, 28).

Secondary, training-related improvements in self-control were studied.

#### Anthropometry

At every time point, patients' height and weight were measured up to the nearest 0.1 cm and 0.05 kg. The BMI was calculated as weight (in kg) over height (in m) squared and further analyzed as the corresponding SDS, adjusted for age and gender based on previously published Flemish growth curves (21).

#### Self-Control Assessments

*1) Bottom-up reactivity: DEBQ: The Dutch Eating Behavior Questionnaire* (29). The DEBQ is a self-report questionnaire that explores bottom-up reactivity in three different maladaptive eating styles within subjects, i.e., emotional eating, external eating, and restraint eating (29). In this study, the focus will be on emotional eating and external eating (eating in reaction to external stimuli). A higher score indicates more maladaptive eating.
*2) Top-down: ECS: Effortful Control Scale* (30). The ECS is a self-report questionnaire that measures top-down self-control. A total effortful control score, as well as two subscale scores can be calculated: “lack of impulsivity” (reflecting general inhibition capacities) and “persistence” (reflecting low distractibility). Only these two subdomains will be further analyzed. A higher score indicates more problems with self-control.
*3) BRIEF: Behavior Rating Inventory of Executive Functioning (BRIEF*) (31), completed by the parents. The BRIEF is developed for assessment of top-down executive functioning as a self-report (for children older than 11), parent or teacher report. It contains a total score as well as 8 subscale scores: inhibition, shifting, emotional control, initiation, working memory, planning/organizing, organizing of materials and monitoring. The first three subscales form the “behavior regulation” index and the last five subscales the “metacognition” index. In this study, only the total score, the inhibition subscale and the behavior regulation factor are used. A higher score indicates more difficulties with executive functioning.

### Statistical Analysis

All statistical analyses were performed with Statistical Package for Social Sciences version 27 (SPSS, NY, USA). Based on a power calculation suggesting an effect size of *f* = 0.10, a total number of 180 patients randomized 1:1 was required to detect a significant effect on BMI with a power of 0.80. A drop-out of 10% was expected, hereby needing 200 participants for inclusion. Due to a higher-than-expected drop-out, 59 additional participants were included (44 inpatient, 15 outpatient).

To detect changes in the outcome variables, i.e., BMI and different domains of self-control, a simple linear mixed model with only time and a random intercept was created. If a significant change over time of the outcome was found, further in-depth analysis was carried out using a second linear mixed model including a random intercept per subject with randomization (self-control or sham), gender, setting (in- or outpatient), and time (T0, T1, T2, T4) as fixed factors and age at baseline and number of sessions (ranging from 1 to 20) as fixed covariates, including all their possible interactions.

The number of sessions was cumulatively expressed and therefore increases over time. Therefore, besides the interaction randomization *by* time, the interaction randomization *by* number of sessions is of particular interest as this incorporates the impact of the increasing number of sessions on the experimental or control group over time (dose–response relation).

Timepoint T3 was excluded from this analysis, as only 20 inpatient participants attended this visit. Patients were excluded from the mixed model analysis if they never completed a single session as otherwise an effect of randomization condition would be assigned although they were never exposed to the self-control or sham training.

For all analyses, a *p*-value < 0.05 was considered significant.

## Results

### Descriptive Statistics

In total, 259 patients were included of which 144 participated in the inpatient and 115 in the outpatient program. The participants from inpatient care were older, had a higher BMI SDS and higher %BMI of the 95^th^ percentile, had a higher score on baseline emotional eating and were more impulsive compared to the patients in the outpatient care, all *p* < 0.001. Both groups were comparable for gender and other self-control-related characteristics (see Table 1A).

**Table 1:** Baseline characteristics of patients in an inpatient setting and patients in an outpatient setting **(A)**, of patients in the sham and patients in the self-control training group **(B)**.

**(A)**	**Inpatient (*n* = 144)**	**Outpatient (*n* = 115)**	***p*-values**
Age (years)	**14.3** **±** **2.2**	**11.9** **±** **2.1**	**<0.001**
♀/♂	84/60	63/52	0.7
BMI SDS	**2.7** **±** **0.4**	**2.4** **±** **0.4**	**<0.001**
%BMI p95	**140.3** **±** **21.0**	**128.4** **±** **16.3**	**<0.001**
ECS: lack of impulsivity	**34.0** **±** **8.0**	**38.1** **±** **6.7**	**<0.001**
ECS: persistence	41.5 ± 8.4	43.5 ± 8.4	0.1
BRIEF: total score	118.9 ± 27.0	117.1 ± 28.5	0.7
BRIEF: inhibition	14.5 ± 4.2	13.7 ± 4.4	0.2
BRIEF: behavior regulation factor	42.7 ± 11.0	41.6 ± 11.4	0.5
DEBQ: external eating	30.9 ± 8.7	28.8 ± 6.2	0.054
DEBQ: emotional eating	**33.0** **±** **14.0**	**24.7** **±** **11.2**	**<0.001**
**(B)**	**Self-control training** **(*****n*** **=** **90)**	**Sham training (*****n*** **=** **86)**	
Age (years)	13.2 ± 2.6	13.0 ± 2.5	0.7
♀/♂	53/33	53/37	0.8
BMI SDS	2.5 ± 0.4	2.5 ± 0.4	0.6
%BMI p95	132.5 ± 16.9	132.0 ± 18.7	0.8
ECS: lack of impulsivity	36.1 ± 6.7	36.0 ± 8.0	0.9
ECS: persistence	43.4 ± 8.4	40.9 ± 8.0	0.1
BRIEF: total score	116.6 ± 28.0	118.6 ± 26.7	0.7
BRIEF: inhibition	13.6 ± 4.1	14.2 ± 4.0	0.4
BRIEF: behavior regulation factor	41.2 ± 11.4	42.2 ± 10.5	0.7
DEBQ: external eating	30.4 ± 8.3	30.0 ± 7.5	0.7
DEBQ: emotional eating	28.3 ± 12.4	32.3 ± 14.4	0.1

*ECS, effortful control scale; BRIEF, behavior rating inventory of executive functioning; DEBQ, dutch eating behavior questionnaire*.

*The values in bold are statistically significant between participants in the in- and outpatient setting*.

There were no differences in baseline age, gender, BMI metrics or self-control between the group with the self-control training and the group with the sham training (see Table 1B).

### Participant Flow

**Figure 3:**
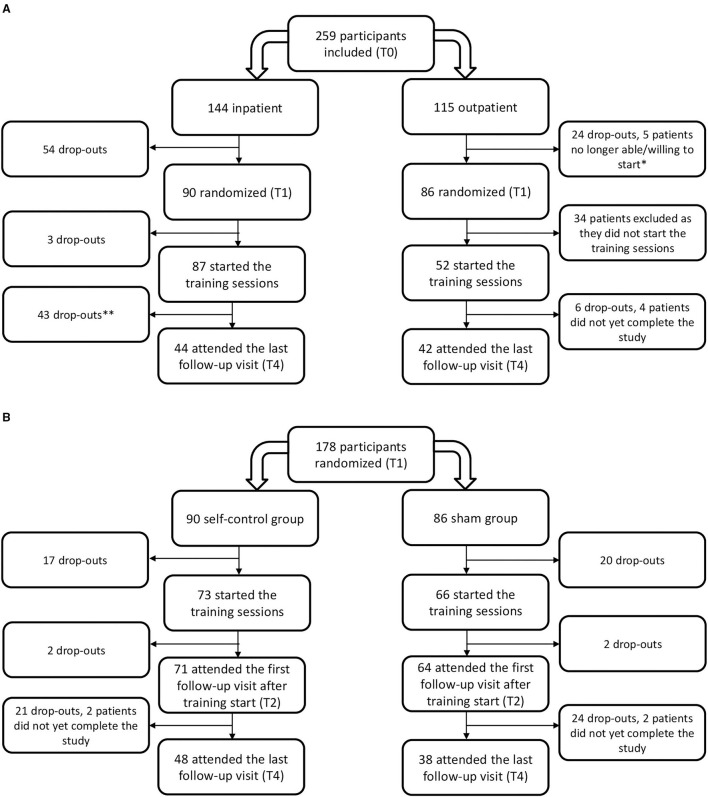
Flowchart of the study participants by **(A)** setting and **(B)** allocated treatment group. *Three participants were admitted to a psychiatric hospital during treatment and two no longer wished to start the training. **All data of patients that dropped-out prematurely or have not yet completed the last visit were included in the analysis until the last documented visit.

The participant flow throughout the study is depicted in Figure 3A.

Inpatient, the highest drop-out was situated before the training started, during the first part of the inpatient treatment program (T0–T1) where 37.5 % (54 out of 144 participants) dropped out. The patients that left the inpatient treatment prematurely did not differ in baseline characteristics regarding age (*p* = 0.3), gender (*p* = 0.3), BMI SDS (*p* = 0.1), or self-control (*p* ≥ 0.1) from those that completed the entire program, except for absolute BMI (38.3 ± 35.6 kg/m^2^ in patients dropping out compared to 35.6 ± 5.6 kg/m^2^ in patients completing the program, *p* = 0.005) and BMI% relative to the p95 (146.3 ± 24.0% compared to 136.4 ± 17.8%; *p* = 0.009). Drop-out at follow-up (T4) resulted in a group of 44 participants that was younger and had a lower obesity severity than those lost to follow-up, witnessed by a mean age of 13.5 ± 2.3 compared to 14.7 ± 2.0 years (*p* = 0.003), and a mean baseline BMI of 37.6 ± 6.4 vs. 34.6 ± 5.3 kg/m^2^ (*p* = 0.003), a mean BMI% of the p95 of 142.8 ± 22.2 vs. 134.9 ± 17.1% (*p* = 0.02) and a mean BMI SDS of 2.6 ± 0.4 compared to 2.8 ± 0.4 (*p* = 0.02). There was no difference in terms of gender and self-control between the 44 participants that completed the entire study compared with those that dropped out prematurely, except for a higher lack of impulsivity score of 32.8 ± 8.2 in the patients that dropped out compared to 36.3 ± 7.0 in the patients that completed the study; *p* = 0.014.

Outpatient, the largest drop-out (39.5%) was found between T1 and T2 at the start of the training sessions, as only 52 out of 86 participants (60.5%) started the training sessions. There was no difference in age (*p* = 0.3), BMI metrics (*p* > 0.4), gender (*p* = 0.8), and self-control (*p* > 0.1) or initial BMI reduction (*p* = 0.6) between the participants that started the training sessions and those that did not, except for more emotional eating in those starting the training compared to those who never trained (27.8 ± 12.7 compared to 22.1 ± 9.0; *p* = 0.042). Of these 52 patients, 42 completed the entire study. These 42 patients were comparable in pretreatment age (*p* = 0.3), BMI metrics (*p* ≥ 0.8), gender (*p* = 0.95) and any domain of self-control (*p* > 0.1) to those that did not start the training or dropped-out before finishing the study.

There was no difference in drop-out between the group with the self-control training and the sham group (*p* = 0.3). Participant flow by randomization condition is shown in Figure 3B.

In the self-control group, the 48 patients attending the last study visit were comparable to the 42 not attending in obesity severity (*p* ≥ 0.6), male-to-female ratio (*p* = 0.3), distribution across treatment settings (*p* = 0.8), and any self-control measures (*p* ≥ 0.2), except age as those not attending were significantly older with a mean age of 13.7 ± 2.6 years compared to 12.7 ± 2.4 years compared to those attending the last study visit, *p* = 0.047. In the sham group, the 38 patients completing the entire study did not differ in age (*p* = 0.1), obesity severity (*p* > 0.1), gender (*p* = 0.5), distribution across treatment settings (*p* = 0.8), or any self-control measures (*p* ≥ 0.2) from the 46 patients not attending the last study visit.

### Training Adherence

In the inpatient center, 97% of patients started with the intensive phase training sessions. After returning home from inpatient treatment, only 19% continued with the boosters. In the outpatient setting, 60% started the intensive phase training sessions, and solely 28% started the booster sessions.

Patients completed on average 7 ± 4 sessions, and there was no difference in number of executed sessions between both settings (7 ± 3 sessions inpatient compared to 8 ± 5 sessions outpatient; *p* = 0.4), nor between both randomization conditions (*p* = 0.1) or based on gender (*p* = 0.9). However, an inverse correlation was found between age and number of sessions (*r* = −0.282, *p* < 0.01). No significant associations were found between any of the self-control variables (measured by the DEBQ, ECS, or BRIEF) and the number of sessions performed (all *p* > 0.2).

### Training Effect on BMI Reduction

**Figure 4:**
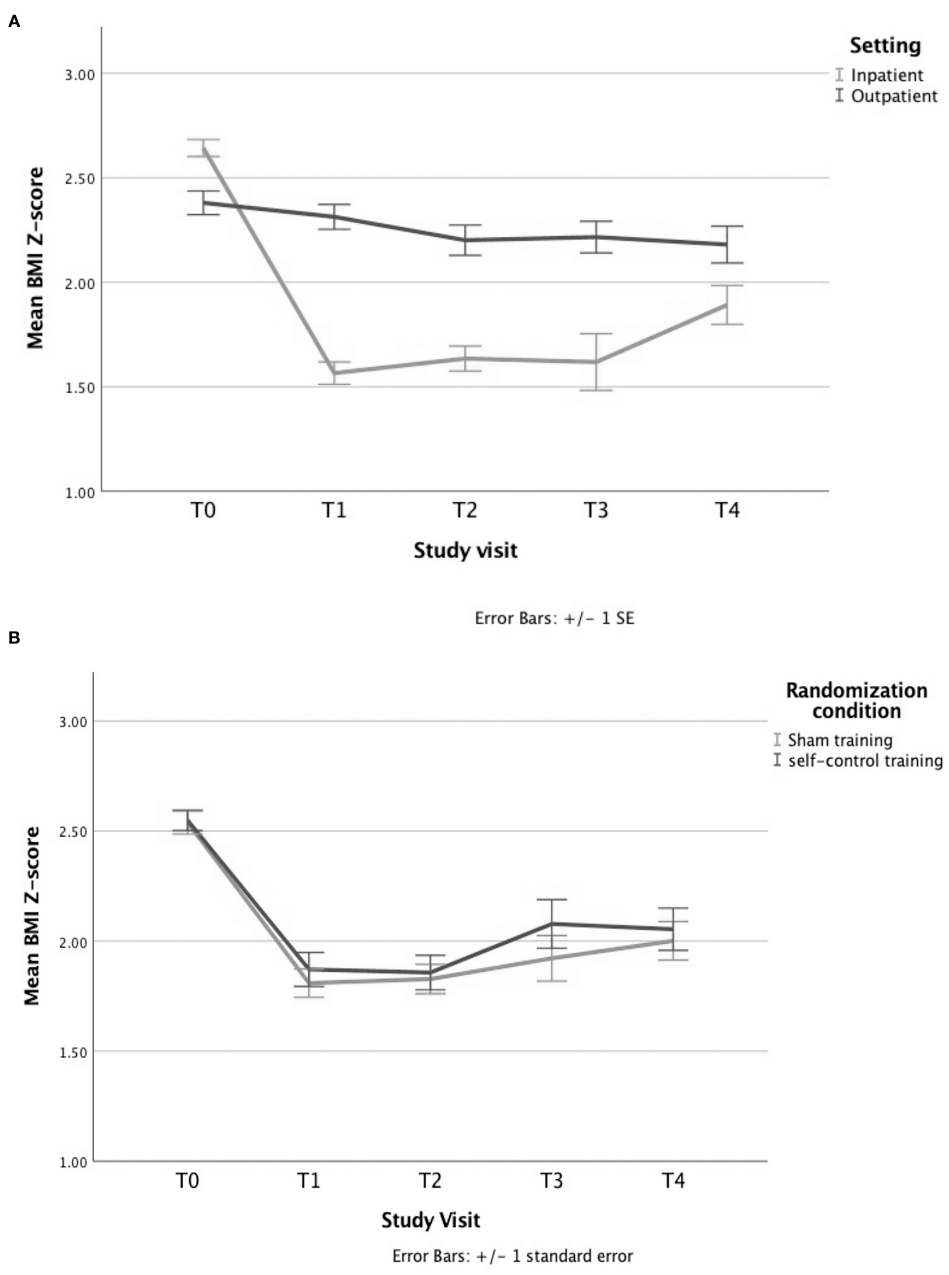
Evolution of BMI Z-score by **(A)** setting and **(B)** randomization condition. **(A)** The BMI decreases significantly over time in both settings, as shown by a simple linear mixed model with a time variable and a random intercept. **(B)** A similar decrease in BMI is seen for the group treated with the sham training vs. those treated with the self-control (SC) training when solely looking at the randomization condition, tested with an independent sample's *t*-test.

In both settings, the participants' BMI decreased significantly throughout treatment as tested with a simple linear mixed model including time only, *p* < 0.001 (see Figure 4A), but no significant differences in BMI could be demonstrated between the sham and self-control condition at any time point (see Figure 4B).

Next, we ran a mixed model analysis on the 139 participants that completed at least one session. The two main interactions of interest were not significant: randomization *by* time (*p* > 0.3) or randomization *by* number of sessions (*p* = 0.1) (see Supplementary Table 1), which was confirmed by analyzing the data as percentage relative to the 95^th^ percentile with *p* > 0.2 for randomization *by* time and *p* = 0.8 for randomization *by* number of sessions (see Supplementary Table 2).

After removal of non-significant terms, a final model was obtained (see Table 2A) containing two significant three-way interactions: randomization *by* number of sessions *by* setting (*p* = 0.002) favoring residential treatment and randomization *by* number of sessions *by* age (*p* = 0.047) favoring younger age, hereby suggesting an effect in a subgroup.

**Table 2A:** Linear mixed model to predict the evolution of BMI SDS over time on the entire population by setting, gender, randomization, time, age, and number of sessions (*n* = 139).

**Parameter**	**Estimate**	**Standard error**	***p*-values**	**95% Confidence interval**	
				**Lower bound**	**Upper bound**
Intercept	2.77	0.68	<0.001	1.44	4.11
Setting	−1.03	0.83	0.2	−2.66	0.60
Gender	**−1.69**	**0.74**	**0.024**	**−3.15**	**−0.23**
Randomization	−0.12	0.42	0.8	−0.95	0.71
T0	0	0			
T1	**−0.10**	**0.039**	**0.011**	**−0.18**	**−0.02**
T2	**−0.18**	**0.048**	**<0.001**	**−0.28**	**−0.09**
T4	**−0.19**	**0.061**	**0.002**	**−0.31**	**−0.07**
Age	−0.036	0.060	0.5	−0.15	0.08
Number of sessions	−0.023	0.019	0.2	−0.06	0.01
Setting * gender	**2.00**	**0.98**	**0.044**	**0.06**	**3.94**
Setting * randomization	−0.06	0.18	0.7	−0.42	0.29
Setting * T0	**0**	**0.05**	**<0.001**	**−1.08**	**−0.88**
Setting * T1	**−0.98**				
Setting * T2	**−0.68**	**0.08**	**<0.001**	**−0.84**	**−0.52**
Setting * T4	**−0.30**	**0.09**	**0.002**	**−0.48**	**−0.11**
Setting * age	0.097	0.07	0.2	−0.04	0.23
Setting * number of sessions	−0.01	0.01	0.3	−0.03	0.01
Randomization * age	0.01	0.03	0.8	−0.06	0.08
Randomization * number of sessions	−0.04	0.03	0.2	−0.09	0.01
Gender * age	**0.15**	**0.06**	**0.025**	**0.02**	**0.27**
Age * number of sessions	0.0014	0.002	0.4	−0.002	0.005
Setting * gender * age	**−0.16**	**0.08**	**0.046**	**−0.31**	**−0.003**
Setting * randomization * number of sessions	**−0.03**	**0.01**	**0.002**	**−0.05**	**−0.012**
Age * randomization * number of sessions	**0.005**	**0.002**	**0.047**	**0.00006**	**0.01**

*Parameters indicated in bold are significant. For factors, one category was used as the reference category and has coefficient 0, whereas the other category has a coefficient, that does not equal 0. This is illustrated in the table by the factor time, where T0 is used as a reference and has coefficient 0, whereas the other timepoints T1–T4 have a coefficient that describes the difference from T0. The documented coefficients in this table apply to the inpatient participants for setting (as compared to the outpatient participants that have coefficient 0), the girls for gender (compared to boys), the group provided with a self-control training for randomization (compared to the sham group). Number of sessions applies to all sessions performed, both the real self-control trainings, as the trainings offered to the control group*.

**Figure 5:**
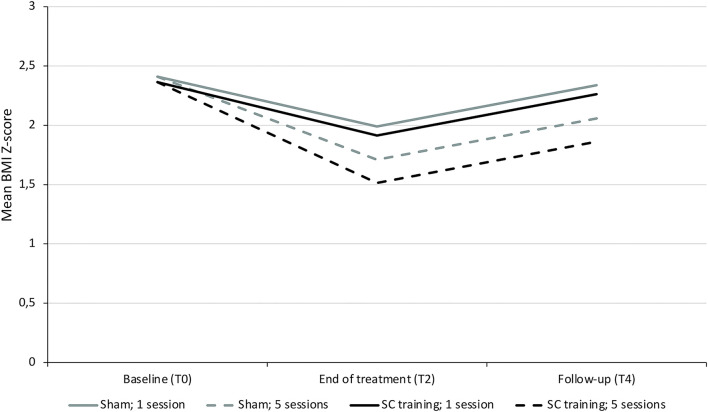
Evolution in BMI Z-score in 8- to 12-year old children treated inpatient in function of randomization and number of sessions. Visual representation of the predicted evolution of BMI Z-score in a subgroup of children aged 8–12 years old treated in residential care. This graph is based on the model in Table 2B, showing the most preferable BMI Z-score evolution in those receiving the most sessions of the self-control training witnessed by the significant interaction of randomization by number of sessions (*p* = 0.027).

An exploratory subgroup analysis indeed confirmed more BMI Z-score reduction in 8- to 12-year-old inpatient-treated children in the self-control group compared to the sham group when considering the dose–response relation, as demonstrated by a significant interaction between randomization and number of sessions (*p* = 0.027, see Table 2B; Figure 5). When repeating the analyses with the BMI percentage relative to the 95^th^ centile, again a positive effect of the number of self-control trainings (interaction randomization ^*^ number of sessions) was found (see Supplementary Figure 1), although this effect was (borderline) non-significant (*p* = 0.08, see Supplementary Table 3).

**Table 2B:** Linear mixed model to predict the BMI SDS evolution over time in a subgroup of 8- to 12- year old children treated in residential care (*n* = 22).

**Parameter**	**Estimate**	**Standard error**	***P*-values**	**Lower bound**	**Upper bound**
				**95% CI**	**95% CI**
Intercept	**2.41**	**0.13**	**<0.001**	**2.14**	**2.68**
T0	0	0			
T1	**−0.97**	**0.072**	**<0.001**	**−1.13**	**−0.83**
T2	−0.35	0.37	0.3	−1.088	0.38
T4	−0.0015	0.40	0.997	−0.81	0.80
Randomization	−0.046	0.18	0.8	−0.41	0.32
Number of sessions	−0.070	0.045	0.1	−0.16	0.019
Randomization * number of sessions	**−0.030**	**0.013**	**0.027**	**−0.056**	**−0.0035**

*Linear mixed model to predict the evolution of BMI SDS over time by randomization condition and number of training sessions. Parameters indicated in bold are significant. For factors, one category was used as the reference category and has coefficient 0, whereas the other category has a coefficient, that does not equal 0. This is illustrated in the table by the factor time, where T0 is used as a reference and has coefficient 0, whereas the other timepoints T1–T4 have a coefficient that describes the difference from T0. The documented coefficient in this table for randomization applies to the group provided with a self-control training for randomization (compared to the sham group). Number of sessions applies to all sessions performed, both the real self-control trainings, as the trainings offered to the control group*.

### Training Effect on Self-Control

To confirm the effect of the training in the younger aged residential participants, we assessed the effectivity of the training and its dose–response effect on self-control in this group. A simple mixed model found no changes in self-control over time, except for persistence and external eating behavior (Table 3). Subsequently, in depth analysis in more complex models was performed for these two outcome variables.

**Table 3:** Evolution of self-control during treatment in the young inpatient participants as tested by a linear mixed model (*n* = 22).

	**T0**	**T2**	**T4**	***p*-values T0 vs. T2**	***p*-values T0 vs. T4**
ECS: lack of impulsivity	36.1 ± 7.6	38.7 ± 6.9	36.2 ± 6.5	0.1	0.6
ECS: persistence	**38.7** **±** **7.8**	**44.1** **±** **6.8**	**42.3** **±** **5.5**	**<0.001**	**0.002**
BRIEF: total score	125 (72–175)	106 (72–152)	116 (94–130)	0.068	0.1
BRIEF: inhibition	13 (10–27)	11 (10–22)	14 (10–17)	0.2	0.3
BRIEF: behavior regulation	38 (28–68)	35 (28–66)	37 (28–52)	0.3	0.4
DEBQ: external eating	**32.8** **±** **10.1**	**23.6** **±** **5.7**	**24.8** **±** **7.2**	**0.001**	**0.001**
DEBQ: emotional eating	23.6 (13–65)	25.5 (13–63)	28.0 (13–50)	0.074	0.061

*EC, effortful control scale; BRIEF, behavior rating inventory of executive functioning; DEBQ, dutch eating behavior questionnaire*.

*Values in bold change significantly over time*.

#### Persistence

The first mixed model with persistence as outcome variable and randomization, gender and time as factors and number of sessions as covariate found no significant effect of randomization *by* time (*p* > 0.1 at every timepoint) or randomization *by* number of sessions (*p* = 0.1) on the evolution in persistence over time. After removal of the non-significant terms, only the effect of time was withheld, *p* < 0.01, as depicted in Table 4A.

**Table 4A:** Linear mixed model analysis to predict the change in persistence (ECS) over time in the subgroup of 8- to 12-year-old children treated in residential care (*n* = 22).

**Parameter**	**Estimate**	**Standard error**	***P*-values**	**95% Confidence interval**	
				**Lower bound**	**Upper bound**
Intercept	38.6	1.5	<0.001	35.5	41.6
T0	0				
T1	5.8	1.5	<0.001	2.9	8.7
T2	5.3	1.4	0.001	2.4	8.2
T4	5.4	1.6	0.001	2.3	8.5

*Final linear mixed model to predict the evolution of persistence (measured by ECS) over time. ECS, effortful control scale. Variables that were non-significant were excluded from the final model*.

#### External Eating

Secondly, a complex mixed model was fitted incorporating the effects of randomization, gender, time and number of sessions. No significant effect of randomization *by* number of sessions or randomization *by* time was found (*p* = 0.8 and *p* ≥ 0.4 at any study visit), so these were removed from the model. A final model for the evolution in external eating is depicted in Table 4B identifying only gender and time as significant contributors of the evolution of external eating with *p*-values of 0.028 and <0.01 respectively.

**Table 4B:** Linear mixed model analysis to predict the change in external eating behavior (DEBQ) over time in the subgroup of 8- to 12-year-old children treated in residential care (*n* = 22).

**Parameter**	**Estimate**	**Standard error**	***P*-values**	**95% Confidence interval**	
				**Lower bound**	**Upper bound**
Intercept	28.6	2.5	<0.001	23.5	33.7
T0	0				
T1	−7.5	2.4	0.003	−12.4	−2.6
T2	−7.9	2.2	0.001	−12.3	−3.5
T4	−8.0	2.3	0.001	−12.7	−3.3
Gender	6.2	2.6	0.028	0.7	11.7

*Final linear mixed model to predict the evolution of external eating (measured by DEBQ) over time*.

*DEBQ, dutch eating behavior questionnaire. Randomization and number of sessions were excluded from the model as these were non-significant. The coefficient reported by gender indicates the difference in external eating behavior for girls compared to boys. Variables that were non-significant were excluded from the final model*.

## Discussion

This is the first multicenter RCT to evaluate the effect of a self-control training on BMI reduction as adjunct to available obesity treatment programs in a large pediatric population across different treatment settings.

In general, the training did not improve BMI outcome in the entire population, unlike previous research conducted by Verbeken et al. reporting better BMI maintenance in the self-control group compared to the control group (32). This previous study used a gamified self-control training, where most participants reported trying to score well on the tasks. In our study, a non-gamified training resulted in major challenges motivating our patients to complete the training sessions, with >25% of the children reporting the training was too demanding (33). As individual engagement is known to impact the efficacy, this might have contributed to the absence of a general effect (34). Another difference is the training tasks: Verbeken et al. used an inhibition and working memory task, whereas we combined an inhibition with an attention task. Possibly, the effect detected previously was explained by the working memory training rather than the inhibition training, as lately the longevity of training effects of the GNG task has been debated (35). Similarly, for the attention task, one adult study reports the 1 week maintenance of the training effect, but evidence on long-term maintenance and that this significantly alters real-life food intake/weight control behaviors could not (yet) be provided (8, 36).

Secondly, an interaction of the “received” dose of the self-control training (randomization *by* number of sessions) with the setting (favoring the inpatient center) was found. The supervision by the staff on the execution of the trainings might have contributed to the effect found only in residential care, as this guaranteed a restriction of environmental distraction and might have motivated to perform the trainings well. In the outpatient setting, caregivers were asked to supervise the training and ensure a quiet environment, however we could not objectify the compliance to these recommendations.

Thirdly, an interaction of the training effect was found with age in favor of the younger aged participants. As younger age was associated with more completed sessions, one could hypothesize that a minimum number of well-executed self-control trainings would be required before an effect can be found. At this moment, the minimal number of sessions necessary to detect an effect is yet unknown and requires further research. A second viable explanation is that the onset of puberty plays a role, as this is accompanied by an oversensitive activation of the reactive brain system, as well as a slower activation of the regulative brain system when confronted with emotional stimuli. This results in a more intense and fluctuating negative affect, which impedes overcoming impulsive behavior (37–39). Therefore, the training might have been more effective in the younger aged group, as these were not (yet) in full puberty.

When looking at training adherence, we notice a high drop-out when patients are asked to train at home. Although our outpatient participation is generally better than in previous e-health studies in this population (40, 41), this remains a major challenge for successful implementation. Preceding adult studies report better participation (15, 42, 43), which can be explained by the inclusion of normal weight subjects (as excess body weight is a risk factor for lower treatment engagement) (15, 43, 44), the offering of external rewards (43) and the patient selection (self-presenting participants specifically for the training vs. children seeking general weight management treatment) (42, 43). Therefore, the present study reflects more truthfully the expected participation when embedding this intervention in clinical pediatric obesity treatment programs and this real-world implementation constitutes an important strength as opposed to the preceding research evaluating almost exclusively the “in-lab effectiveness” of these interventions in “specifically for the training selected” individuals.

Lastly, in the 8- to 12-year-old inpatient participants (where a potential effect of the training on BMI was detected), no effect of the training on self-control was objectified by the questionnaires. A recent review on the mechanisms trained during GNG tasks describes that this task probably solely trains top-down inhibitory control at the very beginning of the training. Thereafter, the effect probably results from an affective devaluation of the no-go cues or possibly from an automatic inhibitory reaction toward these cues (45). As in our study, the questionnaires were filled in after multiple sessions, this might have limited the possibility to detect this improved inhibition. Additionally, questionnaires always carry the risk of recall bias and socially desirable answers (46). Therefore, some choose evaluations filled in by clinicians (47).

Strengths of our study include the multicenter design including a large number of participants distributed across a wide age range, the short- and long-term follow-up data, the evaluation of the training in existing clinical treatment settings and the analyses performed on both BMI SDS and BMI% relative to the p95. Nevertheless, certain limitations should be mentioned. First, there was a considerable drop-out in both settings. To overcome this limitation, an additional 59 patients (15 outpatient, 44 inpatient) were included and a linear mixed model was used for the analysis, as these models are robust against missing data. Coinciding, a low adherence was observed. To address this limitation, we performed the mixed model analysis only on the group completing at least one session, as otherwise an effect of randomization condition was assigned to a participant never experienced an effect of their randomization status. Additionally, the number of sessions completed was incorporated as a covariate in our statistical model to explore the dose–response relation. As previous research already demonstrated beneficial effects on self-reported weight after only four online training sessions in adults with obesity (15), the adjustment for the number of sessions should be considered a strength of our study. Lastly, the presented food cues during the training were not personalized. However, the effect of the GNG training has been found to augment when the presented unhealthy cue was personalized toward the patients' food preferences (48). For the attention training, personalization is already employed in treating anxiety disorders (49) and with conflicting results in social drinkers (50, 51). In obesity research, no evidence is available on whether this enhances the treatment effect, however in the study of Lawrence reporting a positive effect of an inhibition training on self-reported weight specifically selected individuals consuming the no-go snacks.

Looking at the future, researchers are encouraged to first define the optimal training form hereby answering which (combination of) self-control training tasks should be used, which frequency and duration of trainings is preferred and which motivational features are required to make the training attractive and motivating for children, for example by gamification (52), personalizing the presented food cues (48) or adjusting the reward systems to age and gender-related interests (35). Furthermore, although bad adherence results are reported across studies, little research has been devoted to identifying the best implementation strategy for online interventions within this population. As in the present trial setting was identified as a moderator of the training effect, which is probably due to the presence of external control, the role of external supervision on e-health interventions deserves to be further explored. A possible alternative would be to offer the training live by a psychologist as currently tested in adults with obesity (53). Lastly, the availability on smartphones could improve a participants' flexibility on when and how to train (15). However, the impact of these devices on adherence, potency and efficacy remains to be studied as well (15, 42).

In conclusion, the benefit of embedding a computerized self-control training could not be objectified in our overall cohort, but a subgroup of younger children in residential care has potentially benefited regarding BMI reduction when considering the dose–response relation. However, as this finding only applied to a small subgroup, it should first be confirmed in future research. Finally, in future online trainings (and e-health interventions in general), we recommend to first identify suitable implementation strategies ensuring optimal participation, strategies for motivation and accessibility.

## Data Availability Statement

The raw data supporting the conclusions of this article will be made available by the authors, without undue reservation.

## Ethics Statement

The studies involving human participants were reviewed and approved by Ethics Committee of the University Hospital of Antwerp and Ghent (EC n°B670201731779). Written informed consent to participate in this study was provided by the participants and the participants' legal guardian/next of kin.

## Author Contributions

CB, LV, SV, BD, AV, and LB designed the present trial. MY, AV, and EV enrolled the participants and made the database. TN, LV, and CB developed the self-control training. AV did the randomization and invited the participants to start the training sessions. EV, MY, KV, NB, AD, MV, and AT followed the patients clinically, while they were blinded to the treatment condition of the participants (as were the participants themselves). EV, AV, and SV (statistician) performed the statistical analysis and this was reviewed by a second independent statistician. All authors were involved in writing the manuscript and approved the submitted version.

## Funding

Funding was received by the Research Foundation—Flanders: FWO TBM project n°150179, but this organization had no conflict of interest with the results, nor a role in the study design, the collection, analysis and interpretation of the data, the writing of the report or the decision to submit the article for publication.

## Conflict of Interest

The authors declare that the research was conducted in the absence of any commercial or financial relationships that could be construed as a potential conflict of interest.

## Publisher's Note

All claims expressed in this article are solely those of the authors and do not necessarily represent those of their affiliated organizations, or those of the publisher, the editors and the reviewers. Any product that may be evaluated in this article, or claim that may be made by its manufacturer, is not guaranteed or endorsed by the publisher.
