# Supplementary material for: Improving Treatment Outcome in Children With Obesity by an Online Self-Control Training: A Randomized Controlled Trial
Source: Front Pediatr. 2021 Dec 23;9:794256. doi: 10.3389/fped.2021.794256 (PMC8733681; doi:10.3389/fped.2021.794256)
Supplement: Supplementary file 1 [file Data_Sheet_1.docx]

Supplementary Material

| **Table S1: linear mixed model for the evolution of BMI Z-score over time on the entire population including random*time and randomization*number of sessions** | | | | | | |
| --- | --- | --- | --- | --- | --- | --- |
| **Parameter** | **Estimate** | **Standard** | **P-value** | **95% CI** | |  |
|  |  | **error** |  | **Lower Bound** | **Upper**  **Bound** |  |
| Intercept | 2.72 | 0.67 | <0.001 | 1.39 | 4.05 |  |
| Setting | -1.04 | 0.82 | 0.2 | -2.66 | 0.59 |  |
| **Gender** | **-1.64** | **0.074** | **0.028** | **-3.09** | **-0.18** |  |
| Randomization | -0.087 | 0.42 | 0.8 | -0.92 | 0.74 |  |
| T0 | 0 |  |  |  |  |  |
| T1 | -0.092 | 0.047 | 0.053 | -0.18 | 0.0011 |  |
| **T2** | **-0.14** | **0.06** | **0.035** | **-0.26** | **-0.0098** |  |
| **T4** | **-0.15** | **0.08** | **0.07** | **-0.31** | **0.010** |  |
| Age | -0.034 | 0.060 | 0.6 | -0.15 | 0.084 |  |
| Number of sessions | -0.017 | 0.020 | 0.4 | -0.055 | 0.02 |  |
| **Setting * Gender** | **2.01** | **0.98** | **0.042** | **0.074** | **3.94** |  |
| Setting * randomization | -0.070 | 0.18 | 0.7 | -0.42 | 0.28 |  |
| **Setting * T1** | **-0.98** | **0.049** | **<0.001** | **-1.08** | **-0.88** |  |
| **Setting * T2** | **-0.69** | **0.082** | **<0.001** | **-0.85** | **-0.53** |  |
| **Setting * T3** | **-0.30** | **0.095** | **0.002** | **-0.49** | **-0.12** |  |
| Setting * age | 0.097 | 0.067 | 0.2 | -0.036 | 0.23 |  |
| Setting * number of sessions | -0.0091 | 0.012 | 0.4 | -0.032 | 0.014 |  |
| **Gender * age** | **0.14** | **0.064** | **0.028** | **0.016** | **0.27** |  |
| **Gender * number of sessions** | **-0.0097** | **0.0049** | **0.048** | **-0.019** | **-0.00011** |  |
| Randomization * T1 | -0.014 | 0.048 | 0.8 | -0.11 | 0.080 |  |
| Randomization * T2 | -0.081 | 0.078 | 0.3 | -0.23 | 0.073 |  |
| Randomization * T4 | -0.071 | 0.093 | 0.4 | -0.25 | 0.11 |  |
| Randomization * age | 0.0087 | 0.035 | 0.8 | -0.060 | 0.077 |  |
| Randomization * number of sessions | -0.044 | 0.027 | 0.1 | -0.097 | 0.0093 |  |
| Age * number of sessions | 0.00091 | 0.0017 | 0.6 | -0.0024 | 0.0043 |  |
| **Setting * gender * age** | **-0.16** | **0.079** | **0.045** | **-0.31** | **-0.0035** |  |
| **Setting * randomization * number of sessions** | **-0.034** | **0.011** | **0.002** | **-0.056** | **-0.012** |  |
| **Randomization * age * number of sessions** | **0.0058** | **0.0024** | **0.015** | **0.0012** | **0.011** |  |

Parameters indicated in bold are significant. Randomization refers to the treatment allocation to the sham or self-control group. The coefficient in the table represents the difference of the self-control group compared to the sham group. Gender represents the difference for girls versus boys and the coefficient reported by setting refers to the inpatient treatment compared to the outpatient treatment. Number of sessions applies to all sessions performed, both the real self-control trainings, as the trainings offered to the control group. Age corresponds to the age at baseline (T0).

**Table S2: linear mixed model for the evolution of BMI percentage relative to the 95^th^ percentile over time on the entire population including random*time and randomization*number of sessions.**

| **Parameter** | **Estimate** | **Standard. Error** | **P-value** | **95% Confidence Interval** | |
| --- | --- | --- | --- | --- | --- |
|  |  |  |  | **Lower Bound** | **Upper Bound** |
| Intercept | 156.67 | 22.71 | <0.001 | 111.78 | 201.56 |
| Setting | -44.23 | 27.78 | 0.1 | -99.16 | 10.70 |
| **Gender** | **-79.00** | **24.86** | **0.002** | **-128.16** | **-29.85** |
| Randomization | -10.82 | 14.16 | 0.4 | -38.82 | 17.19 |
| T0 | 0 | 0 |  |  |  |
| **T1** | **-4.69** | **1.40** | **<0.001** | **-7.45** | **-1.93** |
| **T2** | **-6.15** | **1.91** | **0.001** | **-9.90** | **-2.40** |
| **T4** | **-6.96** | **2.41** | **0.004** | **-11.70** | **-2.21** |
| Age | -2.49 | 2.0064 | 0.2 | -6.45 | 1.48 |
| Number of sessions | 0.056 | 0.57 | 0.9 | -1.074 | 1.19 |
| **Setting * Gender** | **80.26** | **33.085** | **0.017** | **14.82** | **145.69** |
| Setting* Randomization | -2.46 | 6.053 | 0.7 | -14.43 | 9.51 |
| **Setting * T1** | **-27.86** | **1.47** | **<0.001** | **-30.74** | **-24.97** |
| **Setting * T2** | **-24.93** | **2.44** | **<0.001** | **-29.73** | **-20.12** |
| **Setting * T4** | **-14.15** | **2.83** | **<0.001** | **-19.72** | **-8.59** |
| Setting * age | 4.16 | 2.26 | 0.07 | -0.30 | 8.63 |
| Setting * number of sessions | -0.0051 | 0.34 | 0.98 | -0.68 | 0.67 |
| **Gender * age** | **6.49** | **2.16** | **0.003** | **2.22** | **10.75** |
| Randomization * T1 | -1.14 | 1.42 | 0.4 | -3.93 | 1.65 |
| Randomization * T2 | -2.10 | 2.32 | 0.4 | -6.66 | 2.45 |
| Randomization * T4 | -1.13 | 2.75 | 0.7 | -6.53 | 4.27 |
| Randomization * age | 0.93 | 1.17 | 0.4 | -1.37 | 3.24 |
| Randomization * number of sessions | -0.84 | 0.80 | 0.3 | -2.40 | 0.73 |
| Age * number of sessions | -0.03 | 0.050 | 0.5 | -0.13 | 0.068 |
| **Setting * gender * age** | **-6.55** | **2.66** | **0.015** | **-11.80** | **-1.29** |
| **Setting * randomization * number of sessions** | **-0.78** | **0.32** | **0.017** | **-1.42** | **-0.14** |
| Randomization * age * number of sessions | 0.12 | 0.069 | 0.08 | -0.016 | 0.26 |

Parameters indicated in bold are significant. Randomization refers to the treatment allocation to the sham or self-control group. The coefficient in the table represents the difference of the self-control group compared to the sham group. Gender represents the difference for girls versus boys and the coefficient reported by setting refers to the inpatient treatment compared to the outpatient treatment. Number of sessions applies to all sessions performed, both the real self-control trainings, as the trainings offered to the control group. Age corresponds to the age at baseline (T0).

**Figure S1: Evolution in BMI% relative to p95 in 8- to 12-year old children treated inpatient in function of randomization and number of sessions**

*Visual representation of the predicted evolution of BMI% p95 in a subgroup of children aged 8-12 years old treated in residential care. This graph is based on the model in Table S3, showing the most preferable BMI evolution in those receiving the most sessions of the self-control training, although the interaction of randomization by number of sessions was not significant (p=0.08).*

**Table S3: subgroup analysis in 8- to 12-year-old children in residential care (n=22). Linear mixed model to predict the evolution of BMI percentage of the 95^th^ centile over time by randomization condition and number of training sessions.**

| **Parameter** | **Estimate** | **Standard Error** | **P-value** | **95% Confidence Interval** | |
| --- | --- | --- | --- | --- | --- |
|  |  |  |  | **Lower Bound** | **Upper Bound** |
| Intercept | 127.55 | 3.55 | <0.001 | 120.26 | 134.84 |
| T0 | 0 |  |  |  |  |
| **T1** | **-29.64** | **1.92** | **<0.001** | **-33.47** | **-25.81** |
| T2 | -19.21 | 9.82 | 0.055 | -38.85 | 0.43 |
| T4 | -11.30 | 10.73 | 0.3 | -32.76 | 10.16 |
| Randomization | -2.54 | 4.84 | 0.6 | -12.54 | 7.47 |
| Number of sessions | -1.22 | 1.19 | 0.3 | -3.60 | 1.16 |
| Randomization * number of sessions | -0.62 | 0.35 | 0.08 | -1.32 | 0.08 |

Parameters indicated in bold are significant. For factors, one category was used as the reference category and has coefficient 0, whereas the other category has a coefficient, that does not equal 0. This is illustrated in the table by the factor time, where T0 is used as a reference and has coefficient 0, whereas the other timepoints T1-T4 have a coefficient that describes the difference from T0. The documented coefficient in this table for randomization applies to the group provided with a self-control training for randomization (compared to the sham group). Number of sessions applies to all sessions performed, both the real self-control trainings, as the trainings offered to the control group.

**
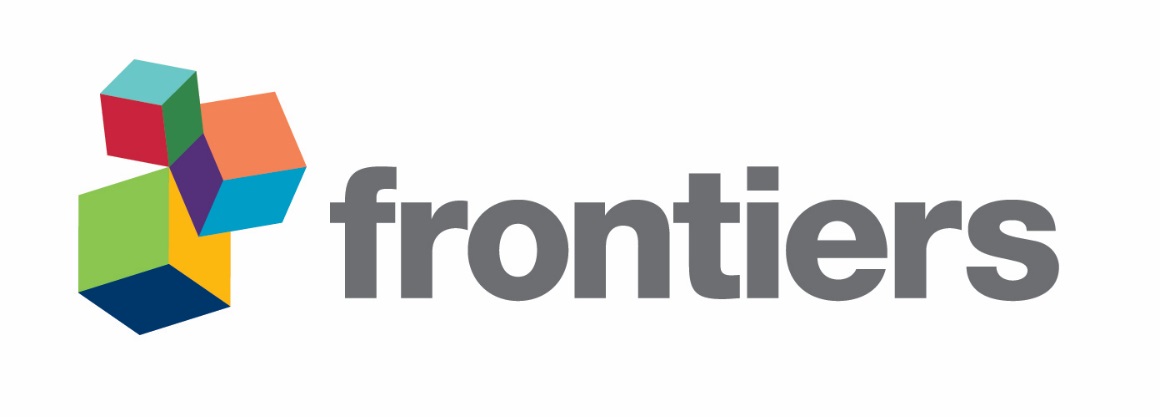
**
